# Use of electronic cigarettes and secondhand exposure to their aerosols are associated with asthma symptoms among adolescents: a cross-sectional study

**DOI:** 10.1186/s12931-020-01569-9

**Published:** 2020-11-16

**Authors:** Abdullah Alnajem, Abdullah Redha, Dalal Alroumi, Ahmed Alshammasi, Mohamad Ali, Maram Alhussaini, Waad Almutairi, Ali Esmaeil, Ali H. Ziyab

**Affiliations:** 1 Faculty of Medicine, Kuwait University, Kuwait City, Kuwait; 2 Department of Community Medicine and Behavioral Sciences, Faculty of Medicine, Kuwait University, Kuwait City, Kuwait

**Keywords:** Electronic cigarettes, Vaping, Combustible cigarettes, Asthma, Respiratory symptoms, Adolescents, Kuwait

## Abstract

**Background:**

Globally, a surge in electronic cigarette (e-cigarette) use has been observed in recent years, with youth being the most susceptible group. Given their recent emergence, studies assessing the health consequences of using e-cigarettes and exposure to their secondhand aerosols (SHA) are limited. Hence, this study sought to assess associations between e-cigarette use and household exposure to SHA from e-cigarettes with asthma symptoms among adolescents.

**Methods:**

A school-based cross-sectional study was conducted by enrolling high school students (n = 1565; aged 16–19 years) in Kuwait. Participants self-completed a questionnaire on tobacco products use (e-cigarettes and cigarettes) and asthma symptoms. Current e-cigarette use and cigarette smoking were defined as any use in the past 30 days. Household exposure to SHA from e-cigarettes in the past 7 days was reported as none (0 days), infrequent (1–2 days), and frequent (≥ 3 days). Asthma symptoms included current (past 12 months) wheeze, current asthma (history of clinical diagnosis and current wheeze and/or medication use), and current symptoms of uncontrolled asthma (≥ 4 attacks of wheeze, ≥ 1 night per week sleep disturbance from wheeze, and/or wheeze affecting speech). Associations were assessed using Poisson regression with robust variance estimation, and adjusted prevalence ratios (aPRs) and 95% confidence intervals (CIs) were estimated.

**Results:**

Among the analytical study sample (n = 1345), current e-cigarette use and cigarette smoking was reported by 369 (27.4%) and 358 (26.6%) participants, respectively. Compared to never e-cigarette users and never cigarette smokers, current e-cigarette users with no history of cigarette smoking had increased prevalence of current wheeze (aPR = 1.54, 95% CI 1.01–2.45) and current asthma (aPR = 1.85, 95% CI 1.03–3.41). Moreover, the frequency of exposure to household SHA from e-cigarettes was associated with asthma symptoms. For example, compared to those with no exposure to household SHA, frequent exposure to household SHA was associated with current wheeze (aPR = 1.30, 95% CI 1.04–1.59), current asthma (aPR = 1.56, 95% CI 1.13–2.16), and current uncontrolled asthma symptoms (aPR = 1.88, 95% CI 1.35–2.62).

**Conclusions:**

E-cigarette use and their household SHA exposure were independently associated with asthma symptoms among adolescents. Hence, such observations indicate that e-cigarette use and passive exposure to their aerosols negatively impact respiratory health among adolescents.

## Introduction

Electronic cigarettes (e-cigarettes; also known as vaping) have rapidly emerged and became the most used tobacco product among youth in many regions of the world. For instance, among high school students in the United States (US), the prevalence of current (past 30-day) use of e-cigarettes increased from 11.7% in 2017 to 27.5% in 2019, making e-cigarettes the most commonly used tobacco product [1, 2]. Similarly, the prevalence of e-cigarette use among adolescents aged 16–19 years in Canada has increased from 8.4% in 2017 to 14.6% in 2018, whereas there was no apparent change in e-cigarette use among adolescents in England (8.7% in 2017 to 8.9% in 2018) [3]. Globally, there is a considerable heterogeneity in the prevalence of e-cigarette use between and within nations, with a general trend of increase in use among youth [4].

The recent surge in e-cigarette use among youth has become a major global public health concern. Reports have shown that e-cigarette use is associated with subsequent use of combustible cigarettes [5–8]; hence, undermining the decades-long progress in reducing smoking among youth [9]. Moreover, observational studies have shown that e-cigarette use is associated with increased risk of marijuana use among adolescents [10, 11]. In regard to acute health effects, the use of e-cigarettes has been linked with a recent outbreak of severe pulmonary injuries and deaths in the US (e-cigarette, or vaping, product use-associated lung injury [EVALI]) that are strongly associated with vaping tetrahydrocannabinol (THC), the main psychoactive compound in cannabis [12, 13].

Aerosols (vapor) emitted by e-cigarettes contain ultrafine particles, such as volatile carbonyls, reactive oxygen species, furans, formaldehyde, and metals (cadmium, lead, nickel, tin, copper, chromium) [14, 15]. Given such constituents, primary or secondary exposure to e-cigarettes aerosols might be associated with health risks. In vitro studies demonstrated that e-cigarette liquids and aerosols cause cytotoxicity in various cell lines, leading to cell death, decreased cell proliferation, and increased oxidative stress [15, 16]. In vivo animal studies showed that inhalation of e-cigarette aerosols lead to airway inflammation, reduced lung function, and impaired innate immunity [15, 16]. In humans, a report showed that airborne nicotine concentrations were higher in homes where e-cigarette use is allowed compared to smoke-free and e-cigarette-free homes, and this observation was further supported by showing that salivary and urinary cotinine levels were higher among non-smokers who were passively exposed to household secondhand aerosols (SHA) compared to non-smokers living in homes with no SHA and secondhand smoke (SHS) exposure [17].

A limited number of population-based studies investigating associations between e-cigarette use and exposure to SHA from e-cigarettes with respiratory symptoms exist [14, 16, 18, 19]. Emerging epidemiological studies have demonstrated positive associations between e-cigarette use and respiratory symptoms [20–30]. A prior study showed that exposure to SHA from e-cigarettes is associated with asthma exacerbations among youth with asthma [31]; however, little is known on whether exposure to SHA from e-cigarettes is associated with respiratory symptoms. Given the scarcity of investigations and the need to better understand the health effects of e-cigarettes, the present study aimed to assess associations between e-cigarette use and exposure to household SHA from e-cigarettes with asthma symptoms (current wheeze, current asthma, and current uncontrolled asthma symptoms) among adolescents.

## Methods

### Study setting, design, and participants

Geographically, Kuwait is divided into six governorates, and the school districts follow a similar geographic distribution. Education in Kuwait is mainly provided by free public schools funded by the state and, to a lesser extent, by private schools. The education system can be divided into four stages, namely, kindergarten, elementary school (1st–5th grade), middle school (6th–9th grade), and high school (10th–12th grade), and in the latter three stages, the students are segregated by sex. Schooling is compulsory for all children aged 6 to 14 years.

This cross-sectional study enrolled schoolchildren (n = 1565) attending public high schools (11th and 12th grade) throughout the State of Kuwait, which included children aged between 16 and 19 years. The schoolchildren were enrolled in the study during the second semester of the 2018–2019 school year (January to May 2019). A stratified two-stage cluster sampling method was used to select a representative study sample of schoolchildren from a random sample of schools. At the time the study was initiated, there were 139 public high schools in Kuwait enrolling approximately 60,663 students (26,692 males and 33,971 females). From a list obtained from the Ministry of Education, Kuwait, of all public high schools stratified by school district and sex, schools were randomly selected using randomly generated numbers. Proportional allocation was used to determine the number of participants needed from each school district by estimating sex-stratified weights relative to the student body size in each given school district. In total, 14 schools served as the recruitment venues for enrolling the required sample size. The study was approved by the Health Sciences Center Ethics Committee for Student Research at Kuwait University (No. 750). Written informed assent was obtained from each participating student. As per the waiver obtained from the Ethics Committee, no consents were sought from the parents.

### Questionnaire and variable definitions

The participants self-completed the study-specific questionnaire that adapted questions from the National Youth Tobacco Survey (NYTS) [2] and the International Study of Asthma and Allergies in Childhood (ISAAC) [32] questionnaires. The questionnaire gathered information on demographic data, e-cigarette use, combustible cigarette use, exposures to SHS and SHA from e-cigarettes, and clinical history and symptoms of asthma.

Never, ever, and current (past 30-day) use of e-cigarettes and combustible cigarettes was ascertained. Participants who reported that they have never used e-cigarettes/combustible cigarettes, even once or twice, were classified as “never users”. Participants who reported ever using e-cigarettes/combustible cigarettes, but not in the past 30 days, were classified as “former users”. Participants who reported using e-cigarettes/combustible cigarettes on ≥ 1 day during the past 30 days were classified as “current users”. Moreover, the frequency of exposure to household SHS and SHA in the past 7 days was assessed by asking: “During the past 7 days, on how many days did someone smoke tobacco products in your home while you were there?” and “During the past 7 days, on how many days did someone smoke e-cigarettes in your home while you were there?” While the frequency of exposure to SHS and/or SHA in public places was assessed by asking: “During the past 7 days, on how many days were you exposed to the smoke of tobacco products or e-cigarettes in public places (e.g., school, restaurants, coffee shops, etc.)?”. These variables were categorized as “no exposure (0 days)”, “infrequent exposure (1–2 days)”, and “frequent exposure (≥ 3 days)”.

Asthma symptoms were defined according to the ISAAC methodology [33]. Current wheezing was ascertained by asking the following question: “Have you had wheezing or whistling in the chest in the past 12 months?” Current asthma (i.e., active asthma in the past 12 months) was defined by an affirmative response to the items “history of doctor-diagnosed asthma” and “wheezing in the past 12 months” and/or “asthma treatment in the past 12 months”. Symptoms of severe asthma included the presence of current wheezing and the occurrence of ≥ 4 wheezing attacks, sleep disturbance from wheezing ≥ 1 night per week, and/or wheezing-affected speech in the past 12 months. Although the ISAAC methodology referred to the aforementioned manifestations as “symptoms of severe asthma”, such features might be better described as “uncontrolled asthma symptoms” that can be considered as signs of more severe disease [34, 35]. Hence, the term “uncontrolled asthma symptoms” was used in this article.

### Statistical analysis

All statistical analyses were carried out using SAS 9.4 (SAS Institute, Cary, North Carolina, USA). The statistical significance level was set at α = 0.05 for all association analyses. Descriptive analyses were carried out to determine the frequencies and proportions of categorical variables. To determine whether the analytical study sample (n = 1345; sample of participants with complete information on status of electronic cigarettes use, cigarette smoking, current wheeze, current asthma, and current uncontrolled asthma symptoms) was representative of the total enrolled study sample (n = 1565), we compared proportions of categorical variables across these two samples using chi-square (χ^2^) tests.

Adjusted associations were assessed by applying a modified Poisson regression with robust variance estimation using the GENMOD procedure in SAS 9.4 to estimate and infer the adjusted prevalence ratios (aPRs) and their 95% confidence intervals (CIs). When assessing associations between e-cigarette use (exposure variable) and asthma symptoms (outcome variable), we further classified never, former, and current e-cigarette users according to their combustible cigarette use status (never, former, and current). Hence, nine mutually exclusive groups were defined: “never cigarette smoker and never e-cigarette use”, “never cigarette smoker and former e-cigarette use”, “never cigarette smoker and current e-cigarette use”, “former cigarette smoker and never e-cigarette use”, “former cigarette smoker and former e-cigarette use”, “former cigarette smoker and current e-cigarette use”, “current cigarette smoker and never e-cigarette use”, “current cigarette smoker and former e-cigarette use”, and “current cigarette smoker and current e-cigarette use”. In the regression analysis, the “never cigarette smoker and never e-cigarette use” group was used as the common reference. In assessing association between frequency of exposure to household SHA from e-cigarettes (exposure variable) and asthma symptoms (outcome variables), the “no exposure (0 days)” was used as the common reference.

## Results

In total, 1575 high school students (732 boys and 843 girls) were invited to participate, and 1565 (729 boys and 836 girls) were enrolled in the study (response proportion: 99.4%). Basic characteristics of the total study sample (n = 1565) and the analytical study sample (n = 1345) are presented in Table 1. The analytical study sample and the total study sample were similar in all characteristics investigated. Of the analytical study sample, 51.8% (697/1345) were females and 53.8% (712/1324) were 17 years old.

**Table 1:** Characteristics of total enrolled study sample and analytical study sample

Variables	Total study sample (n = 1565)	Analytical study sample* (n = 1345)
Sex, n (%)		
Female	836 (53.4)	697 (51.8)
Male	729 (46.6)	648 (48.2)
Age groups, n (%)		
≤ 16 years	386 (25.1)	336 (25.4)
17 years	843 (54.7)	712 (53.8)
≥ 18 years	311 (20.2)	276 (20.8)
Missing, n	25	21
Electronic cigarette use, n (%)		
Never	957 (61.9)	812 (60.4)
Former	182 (11.8)	164 (12.2)
Current (any use in past 30 days)	408 (26.3)	369 (27.4)
Missing, n	18	0
Cigarette smoking, n (%)		
Never	870 (56.5)	743 (55.3)
Former	278 (18.1)	244 (18.1)
Current (any use in past 30 days)	391 (25.4)	358 (26.6)
Missing, n	26	0
Household secondhand aerosol in past 7 days, n (%)		
None (0 days)	1015 (67.9)	882 (67.0)
Infrequent (1–2 days)	144 (9.6)	128 (9.7)
Frequent (≥ 3 days)	336 (22.5)	306 (23.3)
Missing, n	70	29
Household secondhand smoke in past 7 days, n (%)		
No exposure (0 days)	876 (58.1)	758 (57.5)
Infrequent exposure (1–2 days)	146 (9.7)	127 (9.6)
Frequent exposure (≥ 3 days)	486 (32.2)	433 (32.9)
Missing, n	57	27
Current wheeze, n (%)		
Yes	385 (28.1)	381 (28.3)
Missing, n	195	0
Current asthma, n (%)		
Yes	246 (16.1)	242 (18.0)
Missing, n	38	0
Current uncontrolled asthma symptoms, n (%)		
Yes	227 (16.6)	225 (16.7)
Missing, n	195	0

* Refers to a sample of participants with complete information on status of electronic cigarettes use, cigarette smoking, current wheeze, current asthma, and current uncontrolled asthma symptoms (i.e., excluding 220 participants with incomplete information)

Of the analytical study sample, current use of e-cigarettes was reported by 27.4% (369/1345), current combustible cigarette smoking was reported by 26.6% (358/1345), and frequent exposure to household SHA from e-cigarettes was reported by 23.3% (306/1316). In regard to asthma symptoms, prevalence estimates of current wheeze, current asthma, and current uncontrolled asthma symptoms were 28.3% (381/1345), 18.0% (242/1345), and 16.7% (225/1345), respectively (Table 1).

Associations between e-cigarette use and asthma symptoms are shown in Table 2. Compared to the “never cigarette smoker and never e-cigarette use” group, those who are in the “never cigarette smoker and current e-cigarette use” group (i.e., independent effect of e-cigarettes) had statistically significantly increased prevalence of current wheeze (aPR = 1.54, 95% CI 1.01–2.45) and current asthma (aPR = 1.85, 95% CI 1.03–3.41), and a non-statistically significant trend of increased prevalence of current uncontrolled asthma symptoms (aPR = 1.56, 95% CI 0.80–3.06). Hence, these results indicate that current e-cigarette use is associated with asthma symptoms independent of cigarette smoking and other potential confounders (i.e., sex, age, and exposure to household and public places SHS and SHA). Similarly, those who are in the “former cigarette smoker and current e-cigarette use” group had an increased prevalence of the assessed asthma symptoms compared to the reference group. Current cigarette smoking was associated with current wheeze and current asthma independent of e-cigarette use status. For instance, “current cigarette smoke and never e-cigarette use” group (i.e., independent effect of cigarette smoking) had 1.64-fold (95% CI 1.06–2.55) higher prevalence of wheeze compared to the reference group (Table 2). Dual users of e-cigarettes and cigarettes compared to none users of either product demonstrated the strongest associations with asthma symptoms prevalence. For instance, those in the “current cigarette smoker and current e-cigarette use” group had 1.97-times (95% CI 1.35–2.88) increased prevalence of current uncontrolled asthma symptoms compared to “never cigarette smoker and never e-cigarette use” group (Table 2).

**Table 2:** Associations between electronic cigarette use and asthma symptoms according to cigarette smoking status

	Current wheeze		Current asthma		Current uncontrolled asthma symptoms	
Cigarette and e-cigarette use	% (n/total)	aPR* (95% CI)	% (n/total)	aPR* (95% CI)	% (n/total)	aPR* (95% CI)
Never cigarette smoker						
Never e-cigarette use	21.2 (141/665)	1.00 (Reference)	13.4 (89/665)	1.00 (Reference)	11.7 (78/665)	1.00 (Reference)
Former e-cigarette use	30.2 (13/43)	1.30 (0.75–2.23)	16.3 (7/43)	1.43 (0.70–2.92)	14.0 (6/43)	1.34 (0.63–2.86)
Current e-cigarette use	31.4 (11/35)	1.54 (1.01–2.45)	25.7 (9/35)	1.85 (1.03–3.41)	22.9 (8/35)	1.56 (0.80–3.06)
Former cigarette smoker						
Never e-cigarette use	27.2 (28/103)	1.26 (0.89–1.80)	22.3 (23/103)	1.67 (1.08–2.58)	17.5 (18/103)	1.42 (0.88–2.32)
Former e-cigarette use	27.5 (19/69)	1.24 (0.81–1.92)	14.5 (10/69)	1.11 (0.58–2.11)	14.5 (10/69)	1.18 (0.63–2.23)
Current e-cigarette use	33.3 (24/72)	1.52 (1.05–2.21)	20.8 (15/72)	1.71 (1.05–2.78)	27.8 (20/72)	2.28 (1.45–3.58)
Current cigarette smoker						
Never e-cigarette use	36.4 (16/44)	1.64 (1.06–2.55)	20.5 (9/44)	1.73 (1.01–3.21)	13.6 (6/44)	1.01 (0.43–2.38)
Former e-cigarette use	36.7 (18/49)	1.57 (1.02–2.43)	22.5 (11/49)	1.89 (1.05–3.43)	26.5 (13/49)	2.34 (1.38–3.99)
Current e-cigarette use	41.9 (111/265)	1.87 (1.44–2.42)	26.0 (69/265)	1.92 (1.33–2.76)	24.9 (66/265)	1.97 (1.35–2.88)

*aPR* adjusted prevalence ratio, *CI* confidence interval

* Adjusted for sex, age, exposure to household secondhand smoke, exposure to household secondhand aerosols from electronic cigarettes, and exposure to secondhand smoke and/or secondhand aerosols from electronic cigarettes in public places

Associations between frequency of exposure to household SHA from e-cigarettes in the past 7 days with asthma symptoms are presented in Table 3. Frequent exposure (≥ 3 days) to house SHA demonstrated associations with all assessed asthma symptoms. For example, compared to those with no exposure to household SHA, infrequent exposure was associated with a 1.17-times (95% CI 0.87–1.58) higher prevalence of current wheeze, whereas frequent exposure was associated with a 1.30-times (95% CI 1.04–1.59) higher prevalence of current wheeze. Similarly, those who reported frequent household SHA exposure had highest prevalence of current asthma (aPR = 1.56, 95% CI 1.13–2.16) and current uncontrolled asthma symptoms (aPR = 1.88, 95% CI 1.35–2.62) compared to those who reported no household SHA exposure (Table 3).

**Table 3:** Associations between frequency of exposure to household secondhand aerosols (SHA) from electronic cigarettes in the past 7 days and asthma symptoms

	Current wheeze		Current asthma		Current uncontrolled asthma symptoms	
Household secondhand aerosol exposure in past 7 days	% (n/total)	aPR* (95% CI)	% (n/total)	aPR* (95% CI)	% (n/total)	aPR* (95% CI)
No exposure (0 days)	24.1 (215/894)	1.00 (Reference)	14.1 (124/894)	1.00 (Reference)	12.0 (107/894)	1.00 (Reference)
Infrequent exposure (1–2 days)	30.3 (40/132)	1.17 (0.87–1.58)	22.7 (30/132)	1.49 (1.01–2.23)	18.9 (25/132)	1.53 (1.00–2.33)
Frequent exposure (≥ 3 days)	38.8 (120/309)	1.30 (1.04–1.59)	27.2 (84/309)	1.56 (1.13–2.16)	27.8 (86/309)	1.88 (1.35–2.62)

aPR: adjusted prevalence ratio; CI: confidence interval

* Adjusted for sex, age, cigarette smoking status, electronic cigarette use status, exposure to household secondhand smoke, and exposure to secondhand smoke and/or secondhand aerosols from electronic cigarettes in public places

## Discussion

This school-based cross-sectional study aimed to assess associations between e-cigarette use and exposure to household SHA from e-cigarettes with asthma symptoms among a sample of adolescents in Kuwait. The findings indicated that e-cigarette use was associated with increased prevalence of asthma symptoms independent of combustible cigarette use, exposure to household and public places SHS and SHA from e-cigarettes, sex, and age. Moreover, our results showed that frequency of exposure to household SHA from e-cigarettes was associated with asthma symptoms independent of combustible cigarette use, e-cigarette use, exposure to household SHS, exposure to public places SHS/SHA, sex, and age. To our knowledge the finding of associations between exposure to household SHA from e-cigarettes and asthma symptoms is novel. These findings highlight the potential adverse respiratory effects of primary use of e-cigarettes as well as passive exposure to their aerosols among adolescents.

The current study identified positive associations between current e-cigarette use with current wheeze and current asthma that were independent of combustible cigarette use and other covariates. These results are consistent with prior cross-sectional studies that showed associations between e-cigarette use and respiratory symptoms among both adolescents [20–25] and adults [26–29]. The causal inference of such associations is further strengthened by results of a recent longitudinal study that showed e-cigarette use is associated with incident respiratory disease [30]. The effect size of the association between e-cigarette use and current asthma in this report (aPR = 1.85, 95% CI 1.03–3.41) is similar to the effect sizes identified by prior studies, such as reports by Schweitzer et al. (current asthma vs. never asthma: adjusted odds ratio [aOR] = 1.48, 95% CI 1.26–1.74) [20] and Bhatta and Glantz (current asthma vs. no current asthma: aOR = 1.56, 95% CI 1.10–2.22) [30]. Moreover, the highest increase in the prevalence of asthma symptoms was observed among dual users of combustible cigarettes and e-cigarettes, which is in line with previous studies [27, 28]. Hence, we demonstrated that use of e-cigarettes alone associated with increased asthma symptoms, and the burden of these symptoms is further increased when e-cigarette use is combined with combustible cigarette use among the vulnerable population of adolescents. Also, it is important to note that current cigarette smoking showed significant associations with asthma symptoms independent of e-cigarette use status. Hence, further highlighting the established harmful effects of cigarette smoking on respiratory health.

Furthermore, we demonstrated associations between frequency of exposure to household SHA from e-cigarettes with asthma symptoms that were independent of combustible cigarette use, e-cigarette use, exposure to household SHS, exposure to public places SHS/SHA, sex, and age. Although a large body of literature exists on the detrimental health effects of SHS exposure on children and non-smoker adults [36–38], a limited number of population-based studies have assessed the effects of exposure to SHA from e-cigarettes on respiratory health. An experimental study showed that passive exposure to e-cigarette aerosols among non-smoking adults associated with multiple adverse symptoms, including ocular, nasal, and throat-respiratory symptoms [39]. Moreover, Bayly et al. showed that among adolescents with asthma diagnosis, exposure to SHA from e-cigarettes was associated with higher odds of experiencing asthma exacerbations (aOR = 1.27, 95% CI 1.11–1.47) [31]. In the current report we demonstrated that frequent exposure (≥ 3 days in past 7 days) to household SHA from e-cigarettes was associated with increased prevalence of current wheeze, current asthma, and current uncontrolled symptoms of asthma. Up to our knowledge, such results are novel and further highlight the adverse health effects of passive exposure to e-cigarettes aerosols on respiratory health. Given that exposure to SHA is on the rise [40], community-level public health strategies are needed to address e-cigarette use.

Experimental and clinical research have demonstrated the biological plausibility of the effects of e-cigarettes on respiratory health [18]. In vitro experiments have shown that exposing human/animal bronchial epithelial cells to e-liquids/e-cigarettes aerosols is associated with wide array of effects, including altered membrane fluidity, impaired barrier function, increased cell apoptosis, decreased cell proliferation, increased levels of cellular stress, increased cellular toxicity, and increased secretion of inflammatory cytokines [15, 18, 41, 42]. Similarly, animal model investigations showed a range of effects of e-cigarettes, such as increased airway hyper-reactivity; distal airway enlargement; increased lung mucin production, and increased inflammatory cytokines [15, 18, 41, 42]. In humans, exposing healthy never smokers to acute e-cigarette aerosols (i.e., approximately equivalent to smoking 2 combustible cigarettes) was associated with altered biology of lung cells, including the small airway epithelium, alveolar macrophages, and circulating endothelial microparticles [43]. Moreover, a study conducted in apparently healthy men who never used combustible cigarettes showed that e-cigarette use was associated with impaired lung function parameters [44]. Therefore, accumulating evidence indicate that e-cigarette use is not benign and is potentially associated with respiratory disease.

A strength to our study is the enrollment of a school-based random and representative sample of adolescents (11th and 12th grade high school students) from all governorates of Kuwait. Moreover, adapting questions from the standardized NYTS questionnaire allowed us to follow similar definitions used by the NYTS studies [1, 2]. Similarly, adapting questions and definitions used in by ISAAC further strengthened our outcome ascertainments [32, 33]. Nevertheless, our study has some limitations. Although we have used the standardized ISAAC questionnaire, self-reporting of asthma symptoms may introduce information bias. Prior studies have shown that the ISAAC questionnaire has good validity in ascertaining asthma symptoms when compared to physician diagnosis of asthma (sensitivity: 0.85 [95% CI 0.73–0.93], specificity: 0.81 [95% CI 0.76–0.86]) [45] and anti-asthmatic medication reimbursement data (sensitivity: 0.98 [95% CI 0.92–0.99], specificity: 0.98 [95% CI 0.97–0.98]) [46]. Moreover, asthma defined in a similar manner to our “current asthma” variable was reported to be associated with lower lung function parameters [47, 48]; hence, further validating the asthma definition used in this report. The estimated prevalence of current wheeze (28.3%) in this report is similar to a prior estimate of wheeze among children aged 13/14 years in Kuwait (25.9%) [49]. Similarly, our estimated prevalence of current asthma (18.0%) is similar to the prevalence estimate of asthma among 18-year old Isle of Wight birth cohort participants (17.7%), which used a comparable asthma definition [50]. Also, our estimated effect sizes relating e-cigarette use to asthma symptoms are similar to estimates from prior studies [20, 30]. Hence, we anticipate that the effect of information bias, if any, is minimal. Lack of detailed information on adherence to medication among participants with asthma is a further limitation to our study. Moreover, selection bias could also be a concern in cross-sectional studies; however, the possibility of selection bias affecting the results of our study is low because of the high response proportion. Another limitation to our study is the lack of confirmatory information on the extent of household SHA exposure. For instance, information on whether the participants were directly exposed to household SHA (i.e., being in the same room as the e-cigarette user) and the duration of their exposure would have increased the validity of our SHA exposure variable. An inherent limitation of cross-sectional studies is the inability to assess temporal sequence of events. It is also essential to indicate that our analysis aimed to assess concurrent associations rather than to determine temporal and causal associations.

## Conclusions

The findings of this study add to existing knowledge by demonstrating associations between e-cigarette use and exposure to household SHA from e-cigarettes with asthma symptoms among adolescents. The finding of associations between passive exposure to e-cigarettes aerosols and asthma symptoms adds a new perspective and highlights the importance of increasing awareness about the potential harmful effects of such passive exposure, particularly among children and adolescents. Moreover, public health strategies should be placed to curb the ever increasing use of e-cigarettes among adolescents and denormalize and prohibit the use of e-cigarettes in indoor places to reduce passive exposure. Ultimately, comprehensive public health policies and strategies that aim to reduce tobacco use, including e-cigarettes, among adults and adolescents in the population should be developed and implemented.

## Data Availability

The datasets used and analyzed during the current study are available from the corresponding author on reasonable request.

## Abbreviations

E-cigarettes: Electronic cigarettes
SHA: Secondhand aerosol
SHS: Secondhand smoke
CI: Confidence interval
PR: Prevalence ratio
NYTS: National youth tobacco survey
ISAAC: International Study of Asthma and Allergies in Childhood
EVALI: E-cigarette, or vaping, product use-associated lung injury
THC: Tetrahydrocannabinol
